# Special Issue: Improvements for Resveratrol Efficacy

**DOI:** 10.3390/molecules22101737

**Published:** 2017-10-16

**Authors:** Dominique Vervandier-Fasseur, Ole Vang, Norbert Latruffe

**Affiliations:** 1 Institut de Chimie Moléculaire de l’Université de Bourgogne (ICMUB UMR 6302), Université of Bourgogne, 9 Av. Alain Savary, Dijon F-21000, France; dominique.vervandier-fasseur@u-bourgogne.fr; 2 Department of Science and Environment, Roskilde University, DK-4000 Roskilde, Denmark; ov@ruc.dk; 3 Laboratoire de Biochimie (Bio-peroxIL n°7270), 6 Boulevard Gabriel, Université de Bourgogne, Dijon F-21000, France

Resveratrol is a well-known phenolic stilbene because of its presence in several edible plants and its proposed properties that are beneficial to human health. A long list of potential cellular targets for resveratrol have been suggested. However, resveratrol is not a “magical bullet” that we might expect because of delivery problems in humans, at least in part, due to its weak bioavailability. Thus, it is essential to seek new knowledge and different innovating aspects of the development of this multi-targeted natural molecule so that its use for promoting human health food and medicinal preparations can be enhanced. Several exploratory methods, with unexpected natural and original synthetic analogues of resveratrol, in which structural modifications can increase and strengthen biological activities have been considered. Liposomal formulation or encapsulation in cyclodextrins of bioactive molecules might improve their specific delivery; these biotechnological aspects can be applied in the case of resveratrol. Advances in analytical techniques afford an enhanced tracking of resveratrol and its metabolites in cellular environments and the body, and this will lead to a better understanding of the biochemical mechanisms and cellular targets and can therefore provide the basis for new technological achievements. The results of these innovating analyses will provide further knowledge to define a more optimal stilbenoid structure and more optimal delivery systems.

More than a dozen papers have been published upon peer review acceptance (exactly 10 original papers and 4 review papers are included in this issue). They contribute to a better understanding and to advances concerning the improvements for resveratrol efficacy with the following key words: metabolism and bioavailability; natural and synthetic derivatives of resveratrol; biochemical mechanisms; cellular and organ targets of resveratrol and derivatives; analytical techniques. The topics can be summarized as follows:

The improvement of bioavailability and delivery of resveratrol in human was studied by Pentek et al.; they developed a topical resveratrol formulation for commercial applications using dendrimer nanotechnology [1].

Publications of new biotechnological applications for improvements in health, food science and agronomy are presented. Nivelle et al. produced resveratrol and derivatives by grapevine cell suspensions in stirred bioreactor with anti-cancer activity [2], and Kan et al. reported the effects of resveratrol supplementation and exercise training on exercise performance in middle-aged mice [3].

Knowledge of resveratrol for possible application in neurodegenerative diseases is presented. Loureiro et al. prepared resveratrol and grape extract-loaded solid lipid nanoparticles for the treatment of Alzheimer’s disease [4], while Li et al. found that resveratrol ameliorates the depressive-like behaviors and metabolic abnormalities induced by chronic corticosterone injection [5].

Natural derivatives and new synthetic analogues of resveratrol-strengthening biological activities are sought. Chatsumpun et al. evaluated the structural modification and the biological activities of oxyresveratrol [6], while Wang et al. studied the biotransformation of resveratrol by using new prenylated *trans-Resveratrol* synthesized by an *Aspergillus* sp. strain [7].

Advances of analytical techniques for an enhanced tracking of resveratrol and its metabolites in cellular environment are put forward. Dupin et al. performed in vitro glucuronidation and sulfation of ε-viniferin, a resveratrol dimer, in humans and rats [8].

Technological improvements for resveratrol efficacy are performed. Widlund et al. reported that functional mitochondria are important for the effect of resveratrol [9], and Wang et al. showed that resveratrol impacts SIRT1 signaling pathways in a rat model of chronic obstructive pulmonary disease [10].

New and old cellular and tissue targets are characterized for resveratrol and derivatives. Ferraz da Costa et al. [11], and Frazzi & Guardi review cellular and molecular targets of resveratrol on lymphoma and leukemia cells [12], and León et al. review the implications of resveratrol on glucose uptake and metabolism [13]. Finally, Wahab et al. [14] put forward the significance of resveratrol in clinical management of chronic diseases.

The number and the diversity of papers in this special issue on the improvement of resveratrol efficacy confirm the interest in this area. This issue contributes to a better understanding of this attractive compound and its potential implications on biology and health.
